# Determinants of coronary flow reserve in non-diabetic patients with chest pain without myocardial perfusion defects

**DOI:** 10.1371/journal.pone.0176511

**Published:** 2017-04-27

**Authors:** Helena U. Westergren, Erik Michaëlsson, Juuso I. Blomster, Tasso Miliotis, Sara Svedlund, Li-Ming Gan

**Affiliations:** 1 Department of Molecular and Clinical Medicine, Institute of Medicine at Sahlgrenska Academy, University of Gothenburg and Sahlgrenska University Hospital, Gothenburg, Sweden; 2 AstraZeneca R&D, Gothenburg, Sweden; 3 Department of Clinical Physiology, Sahlgrenska University Hospital, Gothenburg, Sweden; 4 Department of Cardiology, Sahlgrenska University Hospital, Gothenburg, Sweden; Medizinische Universitat Graz, AUSTRIA

## Abstract

**Background:**

Microvascular dysfunction could be responsible for chest pain in patients without myocardial perfusion defects. We evaluated microvascular function using ultrasound-assessed coronary flow reserve (CFR) in patients with chest pain and normal myocardial perfusion scintigram. Secondly, we investigated association between cardiovascular parameters and decreased CFR in a sex specific manner.

**Methods:**

A total of 202 (128 women) non-diabetic patients with chest pain and suspected myocardial ischemia, but without myocardial perfusion defects on myocardial perfusion scintigram, were enrolled and underwent CFR examination and blood sampling. All patients were followed-up for cardiovascular events. We used a supervised principal component analysis including 66 variables such as clinical parameters, ongoing medication, coronary artery disease history, lipids, metabolic parameters, inflammatory and other cardiovascular parameters.

**Results:**

During a median follow-up time of 5.4 years, 25 cardiovascular events occurred; (men;18, women;7). Average CFR of the study cohort was 2.7±1.2 and 14% showed impaired CFR<2.0. In an adjusted Cox regression analysis, CFR<2.0 independently predicted event-free survival (HR:2.5, p = 0.033). In the supervised principal component analysis high insulin resistance assessed by Homeostatic model assessment for insulin resistance was the strongest biochemical marker associated with decreased CFR. Interestingly, upon sex specific multivariable linear regression analysis, the association was only significant in men (β = -0.132, p = 0.041) while systolic blood pressure remained an independent predictor in women (β = -0.009, p = 0.011).

**Conclusions:**

In non-diabetic patients with chest pain without myocardial perfusion defects, low CFR has prognostic value for future cardiovascular events. Insulin resistance appears to be a marker for decreased CFR in men. Indeed, in the context of contribution of traditional risk factors in this patient population, the value of systolic blood pressure seems to be important in the women.

## Introduction

Invasive coronary angiography as well as myocardial perfusion scintigram typically identifies obstructive coronary artery disease (CAD) [1]. Coronary microvascular dysfunction (CMD) is likely to also co-exist with obstructive epicardial CAD, as well as being proposed to contribute to the signs and symptoms of ischemia not associated with obstructive CAD [2, 3]. CMD in the absence of obstructive CAD has been shown to be prevalent in patients with chest pain [4] and is associated with an adverse cardiovascular prognosis [5]. Thus, to diagnose and further investigate risk factors associated with CMD, are of emerging importance for clinical cardiology.

CMD is defined as impaired coronary flow reserve (CFR) due to functional and/or structural abnormalities of the microcirculation [2]. Non-invasive transthoracic Doppler echocardiographic assessment of CFR is a reproducible [6] emerging tool to assess the extent of coronary microcirculatory dysfunction [5, 7]. Many parameters besides cardiac function are known to significantly influence CFR including endothelial function, vascular remodeling, vessel density, systemic inflammation and blood viscosity [8]. Microvascular dysfunction is a known pathological phenomenon in diabetes patients and early detection of microangiopathy is a valuable component of vascular risk assessment in type 2 diabetes [9]. In alignment, type 2 diabetes mellitus is associated with increased risk of cardiovascular morbidity and mortality [10] where CMD plays an essential role [11]. Actually, diabetic vs non-diabetic patients with angiographically normal coronary arteries showed decreased CFR [12]. Insulin resistance and the associated compensatory hyperinsulinemia is considered a key mechanism in development of type 2 diabetes, preceding its onset by 10–20 years [13]. In fact, insulin resistance per se, carries prognostic value for future cardiovascular events in subjects without diabetes [14] and has long been associated with impaired vasomotor function [15]. Furthermore, decreased CFR is associated with increased insulin resistance in non-diabetic subjects without coronary angiography verified stenosis, highlighting its potential importance as an early marker of CMD [16].

The presence of microvascular dysfunction has previously been shown more evident in women [17], but recent studies demonstrate similar findings in men [4, 5]. Still, the mechanisms associated with CMD seem differential in men and women [17]. We therefore aimed to study the prognostic power of CFR in non-diabetic patients with chest pain and suspected myocardial ischemia, but without verified perfusion defects on myocardial perfusion scintigram. Further, in patients without inducible myocardial ischemia, we aimed to investigate multiple risk factors relevant for predicting impaired CFR, and hypothesized sex differences in parameters associated with decreased CFR.

## Materials and methods

### Patients and study design

**Fig 1:**
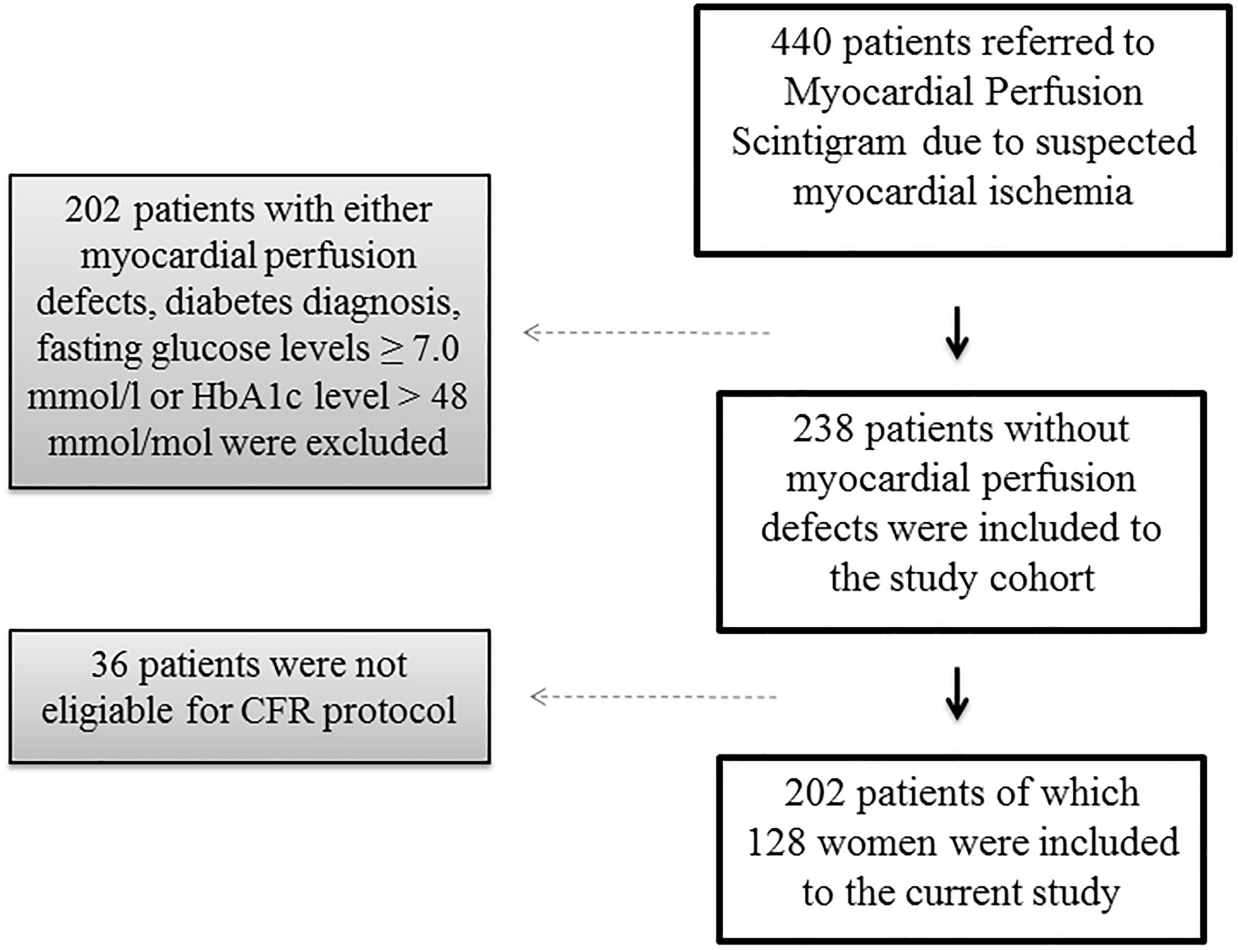
Patient recruitment flowchart over the study population. Flow chart for overview of patient recruitment in the current study. The study was performed at Sahlgrenska University Hospital, Gothenburg Sweden, during the years 2006–2008. CFR = coronary flow reserve.

We performed a prospective observational study in 202 non-diabetic patients with chest pain without myocardial perfusion defects (128 women and 74 men), at the Sahlgrenska University Hospital, Gothenburg, Sweden between 2006 and 2008 (Fig 1).

All patients were referred to myocardial perfusion scintigram due to chest pain and suspect myocardial ischemia, of which 16.8% had previously known CAD. Patients with no sign of ischemic heart disease at the myocardial perfusion scintigram investigation were recruited for study participation within two weeks. All study participants underwent examination of CFR in the left anterior descending coronary artery performed by transthoracic color Doppler guided echocardiography. Previously known CAD was defined as previous coronary artery bypass grafting, percutaneous coronary intervention or myocardial infarction and was collected from patients’ medical records. Blood sampling for laboratory biomarker analysis were carried out after an overnight fast. All patients underwent a standardized interview of demographic data including medical history, smoking status and current cardiovascular medication. All participants provided written informed consent. Inclusion criteria were completed myocardial perfusion scintigram examination due to chest pain followed by transthoracic color Doppler guided echocardiography CFR investigation. Exclusion criteria were myocardial perfusion scintigram-verified ischemia, incomplete CFR investigation, any diabetes diagnosis, fasting plasma glucose ≥7.0mmol/L or glycosylated hemoglobin (HbA_1c_) level >48mmol/mol. All participants provided written informed consent. The study complies with the declaration of Helsinki and was approved by the local Ethics Committee at the University of Gothenburg, Sweden.

### Myocardial perfusion scintigram

Myocardial perfusion scintigram was conducted by a standard clinical two-day protocol. Gated-SPECT studies were performed using ^99m^Tc-sestamibi protocol with non-gated stress and gated rest. Stress test was performed either by symptom-limited exercise test on ergometric bicycle or pharmacological challenge using adenosine infusion. Images were obtained using two different dual-head SPECT cameras (Infinia or Millennium VG, GE Healthcare, Milwaukee, Wisconsin, USA). Reversible myocardial ischemia was observed automatically by the software ECT-tool box. Severity of reversible myocardial perfusion abnormality and extent of ischemia were evaluated by an experienced physician and only patients scored as no sign of ischemia (score 0) on both criteria were recruited for study participation.

### Transthoracic Doppler echocardiography assessed coronary flow reserve

We performed a basic transthoracic echocardiography examination according to current recommendations [18], using Sequoia C256 (Acuson Siemens Mountain View, CA) with a 4V1C transducer. This was followed by transthoracic color Doppler guided echocardiography CFR measurement, where in a modified two-chamber view at the anterior interventricular sulcus, mid- to distal left anterior descending coronary artery was identified using 3.5MHz color Doppler. Flow velocity profiles were registered through pulsed wave Doppler at baseline during rest, and during 5 minutes of adenosine infusion (140μg/kg/min). Electrocardiogram and blood pressure was monitored during the whole procedure. Tracing of mean diastolic flow velocity profile at rest and during peak hyperemia were performed off-line by an experienced physician using the ultrasound software Image Arena (Tomtec, Unterschlissheim, Germany). CFR was calculated as the ratio between hyperemic and basal flow velocity value, and a value <2.0 were considered pathological [7]. Inter- and intra-day variability of CFR assessed by transthoracic color Doppler guided echocardiography has been evaluated by our group in a double-blind randomized clinical trial setting with excellent reproducibility [6]. Reproducibility of transthoracic color Doppler guided echocardiography CFR over a longer time period (3 months) has recently been investigated in healthy volunteers between 35–65 years of age. The coefficients of variation (CV%) in CFR were 3.6% and 4.7% in men and women, respectively.

### Follow-up and definitions of outcome measures

Median follow up-time was 5.4 years (range: 4.6–6.0 years). Follow-up information on outcome was performed by a physician, blinded to the CFR results. Causes of death were examined by data from the Swedish National Board of Health Registry, further follow-up was completed by telephone interviews and confirmed through patient’s medical records. Study endpoint was cardiovascular event-free survival and time to most severe event was analyzed. Cardiovascular endpoint events were defined according to recommendations [19]. Outcome parameters were ranked in the following severity order and defined as the incidence of cardiovascular mortality, nonfatal myocardial infarction/stroke, and coronary arterial revascularizations (either coronary artery bypass grafting or percutaneous coronary intervention). Revascularization was not to be scheduled at myocardial perfusion scintigram investigation. The occurrence of stroke and acute myocardial infarction were collected from patient’s medical records. Myocardial infarction was defined as typical symptoms and pathological troponin changes and/or electrocardiogram changes. Stroke was defined as focal or global neurological deficits lasting for more than 24 hours and verified clinically by a neurologist and/or by computed tomography brain scan.

### Laboratory analysis

All study participants underwent an over-night fasting prior to blood withdrawal. The biochemical analyses were performed using commercially available kits according to the manufacturers' protocols; serum triglycerides and cholesterol (Roche Diagnostics GMBH, Mannheim, Germany), direct high density lipoprotein (HDL) cholesterol (Horiba ABX, France) apolipoprotein A1 (ApoA1) and apolipoprotein B (ApoB) (DakoCytomation, Glostrup, Denmark), cytokines and cell adhesion molecules (Evidence® biochip array technology, Randox Laboratories Limited, United Kingdom), serum insulin (Millipore Corporation, USA). Circulating Interleukin-18 levels were analyzed with an enzyme-linked immunosorbent assay (Medical and Biological Laboratories, Nagoya, Japan). Ultrasensitive Troponin-I was determined using microparticle-based immunoassays and single molecule counting technology (Singulex Ltd, US) generating a LLoQ of 0.4 pg/mL, compared to typical hospital lab tests with a LLoQ at 100pg/mL). Plasma fibrinogen was determined at the Karolinska University Laboratory, University Hospital Solna, Stockhom, by an immunonephelometric method using Polyclonal Rabbit Anti-Human antibody (Q0122, Dako Sweden) and analyzed on Beckman Coulter Immage 800 Immunochemistry System. Plasma Osteopontin was measured using a sandwich enzyme-linked immunoassay (R&D systems). Myoglobin, carbonic anhydrase III, fatty-acid-binding protein, glycogen phosphorylase BB, creatinine kinase MB isoenzyme and cardiac troponin-I were all measured in plasma using the cardiac array panel (Evidence® biochip array technology Randox Laboratories Limited, United Kingdom). Soluble L-selectin, soluble E-selectin and soluble P-selectin were measured in plasma using the adhesion array panel (Evidence® biochip array technology Randox Laboratories Limited, United Kingdom). Vascular endothelial growth factor A and epidermal growth factor in plasma were measured using the cytokine array panel (Evidence® biochip array technology Randox Laboratories Limited, United Kingdom). Plasma myeloperoxidase was analyzed by an enzyme-linked immunosorbent assay (MercodiaAB, Uppsala, Sweden). Plasma glucose was measured using a photometric method and HbA_1c_ by HPLC at the department of Clinical Chemistry, Sahlgrenska University Hospital, Gothenburg. Impaired glucose homeostasis, assessed by homeostasis model assessment of insulin resistance (HOMA-IR) was calculated to estimate insulin resistance using the formula: fasting serum insulin (mU/l) x fasting plasma glucose (mmol/l)/22.5 [20]. L-arginine, symmetric dimethylarginine and asymmetric dimethylarginine in plasma were quantified using isotope dilution mass spectrometry analysis (liquid chromatography coupled to tandem mass spectrometry) using the same instrumentation and methodology as previously described [21].

### Principal component analysis in predicting coronary flow reserve

Principal component analysis (PCA) was performed using Simca 13 (Umetrics, Umeå, Sweden). Before analysis, all data were scaled to unit variance and mean centered. In addition, variables with a max/mean-ratio >10, were log-transformed to increase equal leverage of all variables. This generated a data matrix consisting of 69 variables in 202 patients. To rank and illustrate what variables predicted CFR in this cohort, a supervised PCA was performed. PCA is a projection method that allows for a simultaneous comparison and illustration of the inter-variable relationship and contribution of all variables to an endpoint, in a non-biased manner. The dataset was subjected to an orthogonal projection to latent structures by partial least square analysis (OPLS), in which the variability not related (orthogonal) to what is predicted (CFR in this example) is filtered out [22]. In the model generated by the OPLS, each variable gets a loading score, the amplitude of which quantifies the contribution of the variable to what is predicted in the model. The OPLS analysis was performed to identify and rank 66 variables predicting CFR (basal and hyperemic flow excluded to avoid dominance of these variables in the model). The model described (R2) 21% of the CFR data, with negative predictive (Q2) value of -0.014 after a Jack knife cross validation as implemented in the SIMCA software. Given the biased sex distribution in outcome, an OPLS discriminant analysis was performed to illustrate the major differences between the sexes. The analysis (R2 = 0.58; Q2 = 0.43) indicated a clear separation between the sexes, which justified a sex specific analysis of predictors of CFR.

### Statistics

Deviations in sample size for the various statistical analyses were due to differences in the availability of clinical demographic data, as well as missing values in some analyzed biomarkers/parameters. We calculated the sample size based on the Cox PH one-sided superiority formula. With an overall event rate 15%, an alpha level 5% and 80% power, we need approximately 214 patients to estimate a hazard ratio (HR) of 3.0. Sub-group analysis was performed in a sex specific manner as well as between patients with and without event. The test of skewness was used to assess normal distribution. Demographic data is presented as mean±SD for normal distributed continuous variables and as frequency and percentage for categorical variables. Non-normally distributed variables are presented with their median and interquartile range. Difference between groups was tested using t-test or Mann-Whitney as appropriate, and a p-value <0.05 was considered statistically significant (2-tailed). Categorical data was analyzed by Pearson chi-square test. Kaplan-Meier curves are used to display survival. Hazard Ratio (HR) and 95% confidence intervals (CI) were analyzed by Cox regression models with CFR as categorical variable divided by the pathological cut off <2.0 in both univariate as well as multivariate analysis. Due to the limited number of events (n = 25) in the current study, we selected age and sex based on their known significant clinical value as risk factors to cardiovascular outcome. Furthermore, to study specific sex determinants of the dependent continuous variable CFR, we used multivariable linear regression analysis. Potential covariates tested in the multivariable analysis were selected based on its significant value in the OPLS analysis predicting the dependent parameter as well variables associated with sex specific characteristics. Variables most strongly related to CFR entered the multivariable model with a univariate cut-off value of p<0.250. Possible co-linearity between the x-variables was tested using Spearman correlation coefficient test and a coefficient >0.7 were considered significant. The results from the linear regression analyses are presented as parameter estimates with 95% CIs. All analyses listed above were performed in SPSS (version 21.0, Chicago Inc, USA).

## Results

### Demographic and clinical characteristics

During a time period of two years, 202 non-diabetic patients with suspected myocardial ischemia, but without myocardial perfusion scintigram-verified perfusion defects (128 women and 74 men) were recruited for study participation. Of these, 74% of the patients displayed atypical chest pain, while 26% displayed typical chest pain symptoms in terms of effort angina. Demographic data and history of previously known CAD are presented in Table 1. Briefly, as compared to men, women had higher left ventricle ejection fraction, cholesterol and HDL-levels. Men had higher incidence of previously known CAD and consequently, standard care treatment. Also, increased body mass index and diastolic blood pressure as well as higher levels of glucose, insulin and insulin resistance were evident in men as compared to women. Among the 202 patients, 27 women (21%) and 30 men (40%) were found to have impaired fasting glucose levels (100-125mg/dl; 5.6–6.9mmol/l) as defined by American Diabetes Association. CFR was similar in women and men (Table 1) and the pathological CFR cut off <2.0 was fulfilled by 12% (n = 15) of the women and 19% (n = 14) of the men.

**Table 1:** Baseline characteristics of study cohort.

	Whole study population (n = 202)	Without CV events (n = 177)	With CV events (n = 25)	p-value
Age (years)	62±9	61±9	64±9	0.054
Sex (women)	128	121	7	<0.001
BMI (kg/m^2^, n = 199)	25.3±3.3	24.9±4 (n = 174)	26.5±5	0.024
SBP (mmHg)	143±21	142±21	153±19	0.006
DBP (mmHg)	84±10	84±10	85±13	0.550
Hypertension (n, %)	79 (39.1)	66 (37)	13 (52)	0.158
Current Smoker (n, %)	28 (13.9)	23 (13)	5 (20)	0.251
Previous MI (n, %, n = 196)	18 (8.9)	13 (7) (n = 174)	5 (20) (n = 22)	0.020
Previous PCI (n, % n = 201)	24 (11.9	14 (8) (n = 176)	9 (36)	<0.001
Previous CABG (n, %, n = 201)	11 (5.5)	6 (2) (n = 176)	4 (16)	0.003
Known CAD (n, %)	34 (16.8)	22 (12)	12 (48)	<0.001
LVEF (%, n = 200)	67±11	66±11 (n = 175)	65±8	0.404
Hyperlipidemia (n, %)	85 (42)	71 (40)	14 (56)	0.132
Cholesterol (mmol/L, n = 198)	5.5±1.1	5. ±1.5 (n = 173)	4.9±1.7	0.054
Triglycerides (mmol/L, n = 199)	1.1±0.8	1.1±0.8 (n = 174)	1.3±0.8	0.275
HDL (mmol/L, n = 198)	1.6±0.4	1.5±0.5 (n = 173)	1.2±0.6	0.003
ApoB/ApoA1 (n = 199)	0.7±0.2	0.6±0.2 (n = 174)	0.7±0.2	0.011
Interleukin-6 (n = 196)	1.2±1.2	1.2±1.2 (n = 171)	1.6±1.3	0.043
Fasting Glucose (mmol/L)	5.3±0.5	5.2±0.6	5.5±0.5	0.133
Insulin (mU/L)	13.0±7.4	12.7±6.9	18.2±10.4	0.002
HbA_1c_ (mmol/mol, n = 201)	36.3±3.5	36.2±3.4 (n = 176)	37.0±4.1	0.416
HbA_1c_ (%, n = 201)	5.5±0.32	5.5±0.31	5.5±0.38	0.416
HOMA-IR	3.1±1.8	2.9±1.6	4.0±2.5	0.002
ACE-inhibitors (n, %)	29 (14.4)	26 (15)	3 (12)	0.720
Beta-blockers (n, %)	83 (41.1)	66 (37)	17 (68)	0.003
Statins (n, %)	71 (35.1)	71 (40)	19 (76)	0.005
Aspirin (n, %)	90 (44.6)	56 (32)	15 (60)	0.001

Values are displayed as mean±SD or median and interquartile range for continuous variables and frequency and percentages for categorical variables. ApoA1 = apolipoprotein A; ApoB = apolipoprotein B; BMI = body mass index; CABG = coronary artery bypass grafting CV = cardiovascular; DBP = diastolic blood pressure; HbA_1c_ = glycosylated hemoglobin; HDL = high density lipoprotein cholesterol; HOMA-IR = homeostasic model assessment of insulin resistance; Known CAD = previously known coronary artery disease; LAD = left anterior descending coronary artery; LVEF = left ventricle ejection fraction; MI = myocardial infarction; PCI = percutaneous coronary intervention; SBP = systolic blood pressure.

### Coronary flow reserve predicts cardiovascular outcome in patients with chest pain without myocardial perfusion defects

**Fig 2:**
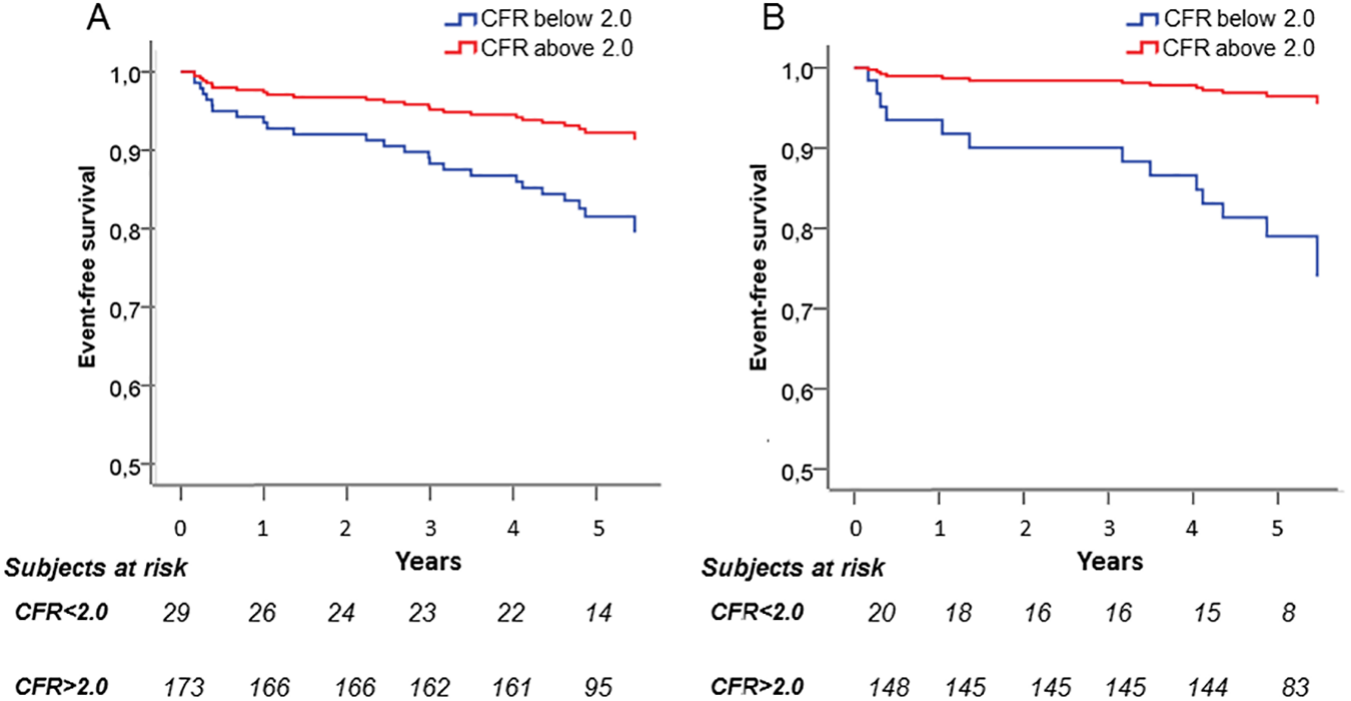
Coronary flow reserve predicts major cardiovascular events in non-diabetic patients with chest pain without myocardial perfusion defects. (A) In a Cox regression analysis, coronary flow reserve below the pathological cut of <2.0 provides an independent prognostic value predicting long-term cardiovascular events in a study population of non-diabetic patients with chest pain without myocardial perfusion defects (n = 202). (B) The prognostic value remains when excluding patients with previously known CAD (n = 168). CAD = coronary artery disease.

Previous studies have shown that CFR <2.0 predicts outcome in patients without obstructive CAD [5, 7]. To validate this in the current patient population, we performed survival analysis and the median follow-up period was 5.4 years (range 4.6–6.0 years). A total of 25 major adverse cardiovascular events, including cardiovascular death (n = 2), acute myocardial infarction (n = 6), stroke (n = 4), elective (n = 6) and emergency (n = 4) coronary percutaneous intervention, as well as elective (n = 3) coronary arterial by-pass grafting, were observed in 12% of the patients (5% (n = 7) in women and 24% (n = 18) in men). First revascularization was performed 60 days after myocardial perfusion scintigram investigation and the median time to event occurrence was 2.4 years. In univariate Cox regression models, CFR <2.0 and sex significantly predicted event-free survival (Table 2). Event rate was significantly higher in patients with CFR <2.0 compared to those with CFR ≥2.0 (27% (n = 8) and 10% (n = 17) respectively, p = 0.007). When adjusting for the traditional cardiovascular risk factors age and sex in multivariable analysis, CFR remained an independent predictor (Table 2). The multivariable adjusted Kaplan-Meier curve is shown in Fig 2A (p<0.001, χ^2^:24.9). The study population consisted of 34 patients with previously known CAD. In a sub analysis, excluding these patients, 13 major cardiovascular events remained. The pathological CFR cut off <2.0 were then fulfilled by 12% (n = 14) of the women and 12% (n = 6) of the men with an event rate of 25% (n = 5) in patients with CFR <2.0 and 6% (n = 8) in those with CFR ≥2.0, p = 0.002. In an over adjusted Cox regression model adjusting for CFR <2.0, age and sex, CFR remained a predictor of event-free survival, alongside sex (Table 2, Fig 2B) (p<0.001, χ^2^:23.2).

**Table 2:** Cox regression analysis. Univariate and multivariable predictors of major adverse cardiovascular events in whole study population and in a subgroup of patients without previous history of coronary artery disease.

	Univariate		Multivariable	
	HR (95% CI)	p-value	HR (95% CI)	p-value
**Whole study population, n = 202**				
Men	4.76 (1.99–11.4)	<0.001	4.59 (1.91–11.0)	0.001
CFR < 2.0	3.19 (1.38–7.40)	0.007	2.52 (1.07–5.91)	0.033
Age	1.04 (0.99–1.09)	0.086	1.04 (0.99–1.08)	0.100
**Patients w/o known CAD, n = 168**				
Men CFR < 2.0	5.40 (1.66–17.6) 5.69 (1.85–17.5)	0.005 0.002	6.04 (1.85–19.7) 6.59 (2.21–20.3)	0.003 0.001
Age	1.01 (0.96–1.08)	0.656	1.02 (0.97–1.08)	0.455

Survival analyses on the study population of non-diabetic patients with chest pain without myocardial perfusion defects. Data are presented with Hazard Ratio and 95% CI. CI = confidence interval; CFR = coronary flow reserve; HR = hazard ratio; Known CAD = previously known coronary artery disease; w/o = without.

### HOMA-IR is the best biochemical predictor for low coronary flow reserve

Reduced CFR per se is a composite biomarker reflecting multiple pathological processes, including flow limiting stenoses, high blood viscosity, increased endothelial adherence of leukocytes and reduced dilatory capacity of resistance vessels (i.e. vascular dysfunction) [8]. These factors, in turn, may have different etiology. To study predictors of reduced CFR in the current cohort, we quantified a diverse set of parameters and biomarkers representing clinical parameters, medication, patient history of CAD, lipids, metabolic parameters together with nitric oxide-pathway linked parameters and other relevant cardiovascular biomarkers including inflammatory- and endothelial parameters (S1 Table).

**Fig 3:**
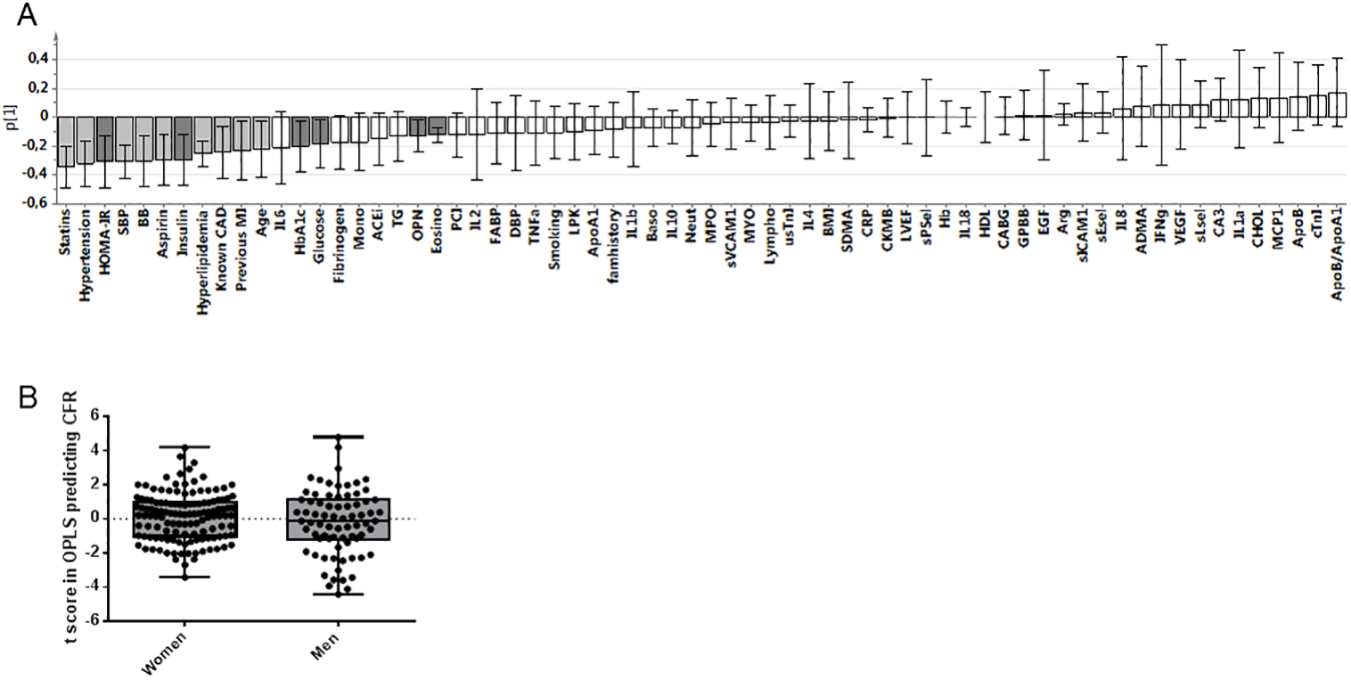
The contribution of variables for explaining CFR-determination. (A) Shows the loadings of the OPLS describing the relationship between the 66 variables and CFR in both sexes (n = 202). Each variable generated a loading value, p(1), along the principal component describing CFR. A positive loading value means that the variable is positively correlated to CFR and vice versa for a negative value. The error bars represent the 95% CI for the variable, and loadings representing variables of which variability is within the 95% CI are highlighted in grey. Dark grey represents plasma blood biomarkers (HOMA-IR, insulin, hyperlipidemia, HbA_1c_, glucose, osteopontin and eosinophil counts). (B) Shows the distribution of men and women along the principal component describing CFR. A high score represents an individual with high CFR and vice versa. The box represents the median and the 25 to 75th percentiles and the whiskers min and max. Each individual is represented by black circle. CFR = coronary flow reserve, HbA_1c_ = glycosylated hemoglobin, HOMA-IR = homeostasic model assessment of insulin resistance, OPLS = orthogonal projection to latent structures by partial least square analysis.

To rank and illustrate which variables predicted CFR in this cohort, a supervised principal component analysis (OPLS) was performed. Fig 3A shows the loading scores of all variables predicting CFR and Fig 3B shows the distribution score of subjects along the principal component describing CFR. As can be seen by the amplitude and direction of the corresponding loadings, the use of statins, hypertension, HOMA-IR, systolic blood pressure (SBP), use of beta blockers, use of aspirin, insulin, hyperlipidemia, previously known CAD, previous myocardial infarction, age, HbA_1c_, glucose, osteopontin and eosinophil counts were all negative predictors of CFR. None of the variables positively correlating to CFR (bars in the positive direction) were considered as significant, as the variability (95% CI) extended through the zero level.

### Discriminant analysis suggests different underlying mechanisms behind coronary flow reserve in men and women

**Fig 4:**
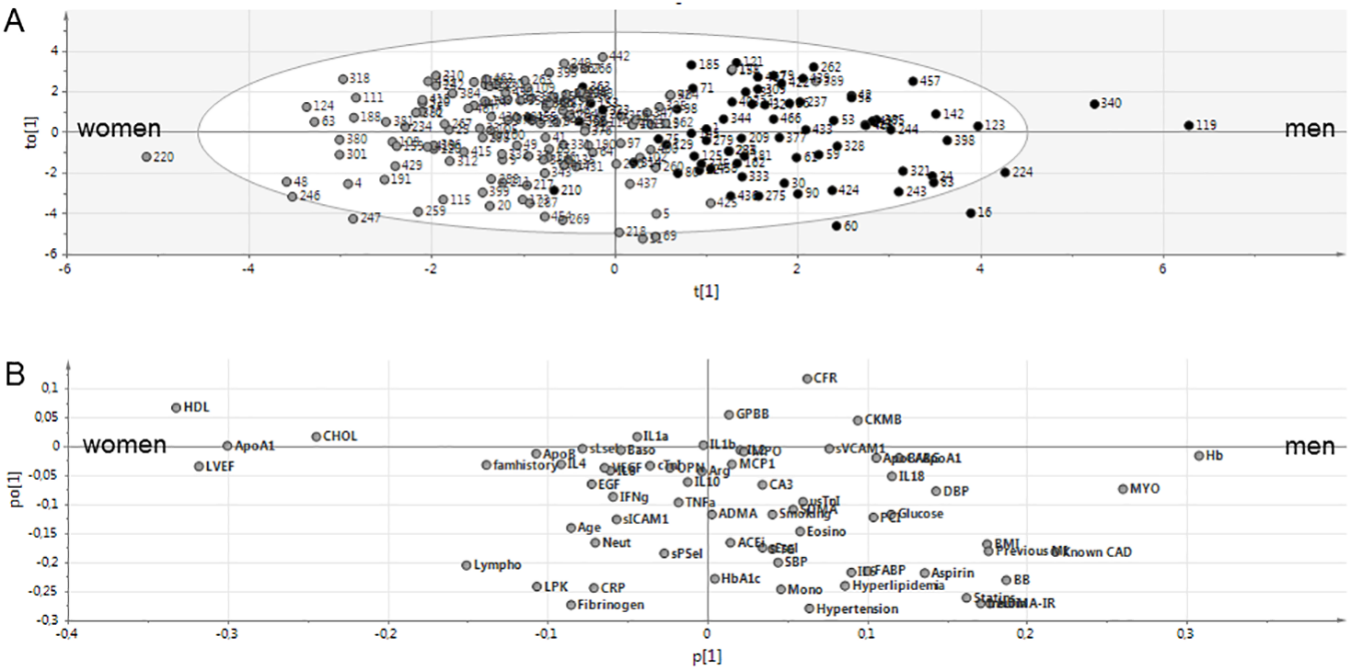
OPLS discriminant analysis illustrating the major differences between men and women. (A) Shows the scores (t, to) of men (black circles) and women (grey circles) based on the loadings (p) and orthogonal loadings (po) of the variables shown in (B). The distribution of loadings along the x-axis ranks the best descriptors for sex differences, whereas the position along the Y-axis ranks the orthogonal loadings i.e. the variablility that is independent of the sex difference. The most pronounced variables (HDL, LVEF, ApoA1, Chol, MYO and Hb) are thus represented by large loading values (in either direction) combined with small orthogonal loading values, and the most outstanding individuals in this comparison is woman #220 and man #119, having the smallest and highest t(1) scores, respectively. The ellipse shown in (A) represents the confidence level (p = 0.05) of the model (n = 202). For explanation of abbreviations, see Table 3. OPLS = orthogonal projection to latent structures by partial least square analysis.

From the distribution of patients along the principal component describing CFR, (i.e. the scores), it appeared that it was a slight skewness in sex distribution (Fig 3B). Although the median and the 25–75% percentiles was similar between the sexes, there were more men among patients having the lowest CFR, arguing that these men may be dominating the OPLS and thereby masking the contribution of the other subjects, including the women. To illustrate the sex differences, an OPLS-discriminant analysis was performed between the men and women. The OPLS discriminant analysis ranks the major differences between two populations and also ranks the non-informative variables, i.e. those variables varying independent (orthogonal) from what is being predicted, which in this example is sex differences. The OPLS discriminant analysis generated a model with a R2 of 0.45 (i.e. explaining 58% of the variability of the sex difference), with a Q2 value of 0.38. Fig 4A shows the distribution (scores) of the subjects in the OPLS discriminant analysis, in which woman #220 and man #119 represent the most extremes. Fig 4B shows the corresponding loading plot that explains the contribution of the variables for the model. From the loading plot, it was evident that the most important (highest/lowest loading values) and robust (smallest orthogonal loadings) variables discriminating the sexes were HDL, cholesterol, ApoA1 and left ventricle ejection fraction, all of which were higher in women than in men, and hemoglobin and myoglobin that were lower in women. The ranking of variables independent of the sex difference are found along the y-axis, highlighting fibrinogen and hypertension as the least informative variable biomarkers discriminating the sexes (close to zero loadings along the x-axis and high (negative) orthogonal loadings along the y axis).

### HOMA-IR and systolic blood pressure are the strongest sex specific parameters predicting coronary flow reserve in men and women, respectively

The differences in the inter-variable relationships in men and women illustrated in the OPLS discriminant analysis, suggested sex specific mechanisms behind CFR and thus we analyzed the data in a sex specific manner, performing a linear regression analysis in respective sex. For the multivariable analysis, co-linearity was found between HOMA-IR and insulin as well as ApoA1 and HDL. HOMA-IR and ApoA1 were selected for further analysis due to greater correlation significance level to CFR in univariate analysis. Upon the sex specific analysis only HOMA-IR remained an independent predictor of CFR in men (Table 3) (R square = 0.13). However, in women only SBP remained an independent predictor of CFR (Table 3) (R square = 0.07).

**Table 3:** Sex specific linear regression analysis. Most associated variables to coronary flow reserve in women and men.

	Univariate		Multivariate	
	β (95% CI)	p-value	β (95% CI)	p-value
**Women, n = 122**				
SBP	-0.010 (-0.016–0.003)	0.006	-0.009 (-0.017–0.002)	0.011
Known CAD	0.374 (-0.144–0.898)	0.155	-0.251 (-0.756–0.254)	0.327
Glucose	-0.219 (-0.538–0.100)	0.177	-0.150 (-0.474–0.174)	0.361
Cholesterol	0.086 (-0.049–0.222)	0.210	0.107 (-0.024–0.238)	0.110
Interleukin-6	-0.018 (-0.047–0.012)	0.236	-0.011 (-0.040–0.018)	0.459
**Men, n = 72**				
HOMA-IR	-0.010 (-0.297–0.041)	0.010	-0.132 (-0.272–0.006)	0.041
ApoA1	-1.245 (-2.267–0.224)	0.018	-0.925 (-1.985–0.135)	0.086
SBP	-0.011 (-0.022–0.001)	0.040	-0.006 (-0.017–0.005)	0.238
Interleukin-6	-0.074 (-0.160–0.012)	0.091	-0.048 (-0.131–0.035)	0.249
Triglycerides	-0.244 (-0.575–0.087)	0.146	-0.073 (-0.407–0.261)	0.665

Linear regression analyses on non-diabetic patients with chest pain without myocardial perfusion defects. ApoA1 = apolipoprotein A1; CI = confidence interval; Known CAD = previously known coronary artery disease; SBP = systolic blood pressure.

## Discussion

In the current study we present CFR as a prognostic marker predicting adverse cardiovascular events in non-diabetic angina patients with chest pain and suspected myocardial ischemia, but without myocardial perfusion defects. In a more detailed analysis, we investigated the association of CFR to multiple relevant cardiovascular parameters and biomarkers where insulin resistance measured by HOMA-IR was the strongest biochemical marker predicting decreased CFR. To address our hypothesis we performed a sex specific linear regression analysis investigating predictors of CFR, which yielded HOMA-IR as to be associated with decreased CFR in men, and SBP in women. These results highlight the importance of addressing suspected CMD in angina patients without myocardial perfusion defects, and where emphasis on risk factors such as HOMA-IR and SBP can guide clinical care in a sex specific manner.

### Microvascular dysfunction and coronary flow reserve

Microvascular dysfunction in the absence of obstructive CAD has gained increasing attention and CFR has become one emerging method to detect and quantify CMD [2, 7]. CFR can be assessed with transthoracic ultrasound alongside [23] other noninvasive methods including magnetic resonance imaging and positron emission tomography [24], but in comparison to these, ultrasound offers a non-radioactive and cost efficient option with excellent reproducibility in experienced hands [6]. In absence of obstructive CAD CFR in left anterior descending coronary artery is considered to reflect global microvascular dysfunction and has recently been recommended in ESC guidelines 2013 [25]. In addition, we have previously shown that transthoracic color Doppler guided echocardiography CFR measured in any of the three epicardial coronary arteries predicted significant CAD independently of anatomical localization and number of diseased arteries determined by angiography [26]. When CAD advances, the regulation of coronary blood flow is disturbed in the whole heart and not only in the most diseased vessel. Therefore one could limit the examination to left anterior descending coronary artery since it is technically less demanding, less time-consuming, and therefore more patient-friendly. Commonly occurring hallmarks of myocardial ischemia, such as stress-induced ventricular wall motion abnormalities, are typically absent in patients with microvascular dysfunction [4]. Indeed, assessment of CFR provides additional prognostic information in subjects with suspected coronary artery disease despite negative dipyridamole stress echocardiography [27]. In addition, impaired CFR has been shown to predict cardiovascular mortality in non-diabetic patients referred to cardiac positron emission tomography, without myocardial perfusion defects [28]. In the current study, none of the patients displayed myocardial perfusion defects or obvious wall motion abnormalities as detected by myocardial perfusion scintigram. In agreement with previous findings, we demonstrate impaired CFR is associated with worse prognosis in chest pain patients without inducible myocardial ischemia regardless of previous known CAD history.

### Predictors of coronary microvascular function and coronary flow reserve

The mechanisms involved in microvascular dysfunction can be induced by e.g. hypertension, hypercholesterolemia, diabetes and obesity [29]. Therefore, we analyzed multiple relevant variables possibly predicting adenosine-induced CFR in our cohort. The clinical risk factors associated with decreased CFR in our study were previous history of CAD and consequently current medical treatment as well as hypertension, SBP, hypercholesterolemia and age, confirming a relation between CFR and an unfavorable clinical phenotype for CMD [3, 30]. However, these traditional risk factors could explain CMD only to a limited extent [4], thus additional biomarkers are considered necessary to improve risk stratification [3, 30]. Therefore, we analyzed several biochemical markers relevant for CMD. In a descending order, increased HOMA-IR, insulin, HbA_1c_, glucose, osteopontin and eosinophils were the biomarkers predicting decreased CFR, emphasizing the value of impaired glucose homeostasis in this population. In agreement, insulin resistance has been shown to be associated with decreased CFR in non-diabetic patients with chest pain and non-obstructive CAD [16] as well as in first degree relatives of type 2 diabetic patients [31]. Our study demonstrates the significance of insulin resistance in CMD also in chest pain patients without inducible myocardial ischemia Interestingly, although the roles of osteopontin and eosinophils seem of less importance, both are relevant for CMD. Osteopontin levels have been shown to correlate with the extent of PET-verified coronary microvascular dysfunction [32], while eosinophils have been suggested to play a role in endothelial damaging and pro-thrombosis in coronary heart disease [33]. Taken together, impaired glucose tolerance and insulin resistance in particular might add value to traditional risk markers in risk stratification of CMD in non-diabetic patients with chest pain without myocardial perfusion defects.

### Sex specific effects

Non-obstructive CAD is more prevalent in women than men with stable ischemic heart disease [34]. However, it was recently shown that the prevalence of microvascular dysfunction in patients with chest pain and non-obstructive CAD is high in both men and women using invasive CFR [4], and CFR<2.0 predicts major adverse cardiovascular events in both sexes [5]. The underlying cause of CMD in women and men seems to be distinct [17] and adjusting for sex may not encounter sex differences. Interestingly, in a sex specific regression analysis HOMA-IR was the strongest predictor of CFR in men, whereas in women SBP independently predicted CFR. HOMA-IR incorporates both glucose and insulin concentrations and shows stronger association with cardiovascular disease than glucose or insulin concentration alone in non-diabetic patients [14]. Insulin is a vasodilator at the arterial, venous, and microcirculatory levels [35, 36] and insulin resistance has been shown to be related to coronary microvasculature abnormalities in patients without diabetes [16], suggestive of an important underlying mechanism in onset of this disease [37]. Indeed, microvascular dysfunction in terms of retinopathy, nephropathy, and peripheral neuropathy are common pathological findings in patients with diabetes. Thus, an association between insulin resistance in non-diabetic patients and CMD is of relevance [3].

Supporting the role of SBP finding in women hypertension is known to influence the underlying mechanisms in microvascular dysfunction [29] and induce coronary structural and functional alterations [2, 30]. Hypertension in women is often undiagnosed or inadequately treated [38] emphasizing the value of assessing SBP in addition to hypertension diagnosis in female CMD. Indeed, the Women's Ischemia Syndrome Evaluation (WISE) study showed SBP alongside CFR to be of significant value in predicting cardiovascular outcome in women with absence of coronary artery stenosis warranting coronary revascularization [39]. In the WISE study, traditional risk factors were associated with CMD, but only accounted for <20% of the observed variability [40]. Also in this study we observe low variability for determination of CFR in women, suggesting further parameters than the many observed here to probably be of importance. However, in alignment with our results, Sade et al. demonstrated that pathological CFR was more pronounced in hypertensive women [41]. In summary, our results indicate that insulin resistance in men is an important parameter contributing to impaired CFR in the current cohort, possibly reflecting coronary microvascular dysfunction. Interestingly, SBP was a significant predictor of CFR in women in this patient cohort.

### Study limitations

The current study is an observational study and maybe subjected to the limitations associated with sample size, unadjusted confounding factors and missing data. The authors encourage caution in interpreting data regarding survival analysis due to the low even-rate. Furthermore, body mass index was used instead of waist circumference or epicardial fat thickness which could have contributed with more specific information regarding body fat distribution; a known cardiovascular risk factor. We have included patients with and without previous CAD supported by the fact that all had normal myocardial perfusion scintigram results. We further presented data separately in patients without CAD in the outcome prediction model. However, to further dissect the cohort into with and without known CAD and specific sex analysis will make the OPLS-PLA model and sex specific linear regression models underpowered. In the adjusted sex specific linear regression analysis, SBP and HOMA-IR remain as predictors of CFR in women and men respectively, although with borderline significant p-value in men. The data therefore needs to be confirmed in larger studies holding higher statistical power than the current study. Finally, the current study is of limited sample size, caution should be taken when interpreting the data.

### Conclusions

In conclusion, in non-diabetic patients with chest pain and suspected myocardial ischemia, but without myocardial perfusion defects, addressing CFR seems to provide an additional tool for cardiovascular risk prediction. However, there appears to be sex specific differences whereas in men insulin resistance seems of significance contributing to decreased CFR. Indeed, in the context of traditional risk factors, the value of SBP appears to be important in women.

## Supporting information

S1 Table Parameters used in OPLS analysis for prediction of coronary flow reserve. (DOCX)

S1 Minimal Dataset (XLSX)

## Abbreviations

ApoA: apolipoprotein A
ApoB: apolipoprotein B
CAD: coronary artery disease
CFR: coronary flow reserve
CI: confidence interval
CMD: coronary microvascular dysfunction
HR: hazard ratio
HbA_1c_: glycosylated hemoglobin
HDL: high density lipoprotein cholesterol
HOMA-IR: homeostasic model assessment of insulin resistance
OPLS: orthogonal projection to latent structures by partial least square analysis
PCA: principal component analysis
SBP: systolic blood pressure
