# Supplementary material for: Determinants of coronary flow reserve in non-diabetic patients with chest pain without myocardial perfusion defects
Source: PLoS One. 2017 Apr 27;12(4):e0176511. doi: 10.1371/journal.pone.0176511 (PMC5407821; doi:10.1371/journal.pone.0176511)
Supplement: S1 Table — (DOCX) [file pone.0176511.s001.docx]

**S1 Table Parameters used in OPLS analysis for prediction of coronary flow reserve**

| **Clinical parameters**  Age  Body Mass Index (BMI)  Systolic blood pressure (SBP)Diastolic blood pressure (DBP)  Smoking  Left ventricle ejection fraction (LVEF)  Hemoglobin (Hb)  Hypertension  Hyperlipidemia | **Inflammatory and other cardiovascular parameters**  C-reactive protein (CRP)  Interleukin-1α (IL1a)  Interleukin-1β (IL1b)  Interleukin-2 (IL2)  Interleukin-6 (IL6)  Interleukin-8 (IL8)  Interleukin-10 (IL10)  Interleukin-18 (IL18)  Tumour necrosis factor-α (TNFa)  Interferon-γ (IFNg) | **Nitric oxide pathway**  L-Arginine (Arg)  Symmetric Dimethylarginine (SDMA)  Asymmetric dimethylarginine (ADMA) |
| --- | --- | --- |
| **Medication**  Angiotensin-converting-enzyme inhibitor (ACEi)  Aspirin  Betablockers (BB)  Statins | Monocyte chemotactic protein 1 (MCP1)  B-leukocyte particle concentration (LPK)  Monocytes (Mono)  Neutrophiles (Neut)  Lymphocytes (Lympho) |  |
| **Coronary artery disease history**  Previously known coronary artery disease (Known CAD)  Previous myocardial infarction (Previous MI)  Previous percutaneous coronary intervention (PCI)  Previous coronary artery by-pass grafting (CABG)  Family history of coronary artery disease (famhistory) | Basophiles (Baso)  Eosinophiles (Eosino)  Myeloperoxidase (MPO)  Myoglobulin (MYO)  Carbonic anhydrase III (CA3)  Fatty-acid-binding protein (FABP)  Glycogen phosphorylase BB (GPBB)  Creatine Kinase MB Isoenzyme (CKMB)  Cardiac troponin-I (cTnI)  Ultra-sensitive cardiac troponin-I (usTnI) |  |
| **Lipids**  Cholesterol (Chol)  Triglycerides (TG)  High density lipids (HDL)  Apolipoprotein A1 (ApoA1)  Apolipoprotein B (ApoB)  Apolipoprotein B /Apolipoprotein A1 (ApoB/ApoA1) | Soluble L-selectin (sLsel)  Soluble E-selectin (sEsel)  Soluble P-Selectin (sPsel)  Vascular endothelial growth factor A (VEGF)  Epidermal growth factor (EGF)  Fibrinogen  Osteopontin (OPN)  Soluble vascular cell adhesion molecule 1 (sVCAM1) |  |
| **Metabolic parameters**  Insulin  Glucose  HbA_1c_  HOMA-IR | Soluble intercellular adhesion molecule 1 (sICAM1) |  |
